# Effect of aerobic and resistance training on body composition, functional capacity, and quality of life in patients with liver cirrhosis: a systematic review with meta-analysis

**DOI:** 10.1016/j.clinsp.2025.100748

**Published:** 2025-09-20

**Authors:** Thiago Casali Rocha, Rafael Ribeiro Germano, Maria Clara Dias Giacomini, Fábio Heleno de Lima Pace, Mateus Camaroti Laterza, Daniel Godoy Martinez

**Affiliations:** a Cardiovascular Research Unit and Exercise Physiology, Federal University of Juiz de Fora, Brazil; b Division of Gastroenterology and Hepatology Service, Department of Medicine, University Hospital, Federal University of Juiz de Fora, Juiz de Fora, MG, Brazil

**Keywords:** Liver cirrhosis, Aerobic exercise, Resistance exercise, Quality of life, Exercise therapy

## Abstract

**Purpose:**

Evaluate the effects of resistance and aerobic training on the quality of life (QoL), functional capacity, and body composition of patients with liver cirrhosis.

**Methods:**

Studies published in the MEDLINE database via PubMed, Cochrane Library, and Web of Science were analyzed. Patients with liver cirrhosis who performed aerobic or resistance exercises in the experimental group and controlled and randomized clinical trials (RCTs) were included, whereas publications with only abstracts, control groups that performed aerobic and/or resistance training, and studies that included cointervention in the experimental and control procedures were excluded. Study variables were QoL, VO2peak, body mass index (BMI), distance covered in the 6-minute walk test (6 MWT), and thigh circumference. PRISMA systematization was used to prepare this review.

**Results:**

Six RCTs were included in this review, including 244 participants aged 53–63 years with Child–Pugh stages A, B, or C, and mostly men. With QoL as a continuous variable, the difference in standardized means was 0.36 [0.01 – 0.76]; *p* = 0.04 for studies that performed resistance training. With 6 MWT as a continuous variable, significant improvement was seen after resistance and aerobic training, and the difference in means was 49.39 [16.53 – 82.24]; *p* = 0.03. No difference was observed after performing aerobic and resistance exercises in relation to VO2peak, thigh circumference, or BMI (*p* > 0.05). Certainty of evidence assessed by the GRADE was between very low and moderate.

**Conclusion:**

Aerobic and resistance physical training appear to improve the QoL and functional capacity without changing body composition in patients with liver cirrhosis.

## Highlights

• First meta-analysis comparing aerobic and resistance training in liver cirrhosis.
• Resistance training seems to improve the quality of life in liver cirrhosis.
• Aerobic and resistance training should be part of cirrhosis treatment.

## Introduction

Liver cirrhosis is caused by chronic and permanent damage to the liver parenchyma. It is characterized by nodules and diffuse fibrous bands and may have various etiologies.^1^ The number of cases has increased significantly worldwide over the last decade, accounting for 2.4 % of all deaths worldwide.^2^ People over 50-years of age, men, and those with metabolic syndrome are most affected by the disease.^3^

Cirrhosis is usually characterized by a long latency period and no significant clinical manifestations, making early diagnosis difficult and allowing the disease to progress until complications develop.^4^ The prognosis is highly influenced by the underlying etiology, with alcoholic cirrhosis generally presenting with a more rapid progression. Early therapeutic intervention based on hepatic biochemical markers and imaging can slow disease progression and improve patient survival rates.^5^

Therefore, it is essential to evaluate the predictors of decompensation during the treatment of liver cirrhosis, such as the presence or absence of portal hypertension, albumin levels, and Model for End-Stage Liver Disease (MELD) score,^6^ to avoid events such as gastroesophageal varices, ascites, and hepatic encephalopathy.^7^

In an outpatient setting, the assessment of muscle strength, specifically handgrip strength, frequently reveals sarcopenia in patients with liver cirrhosis. This simple and objective measurement is a reliable predictor of disease decompensation and is associated with a higher risk of mortality.^8^ Notably, due to hypermetabolism and the decline in protein intake and absorption,^9^ liver cirrhosis predisposes patients to develop sarcopenia,^10^^,^^11^ which, in turn, is directly related to worsening quality of life^12^ and more than two times increased mortality risk.^13^

To significantly reduce the loss of muscle mass and improve the quality of life of patients with liver cirrhosis, nonpharmacological measures such as a high-protein diet^14^ and physical training^15^ have been investigated. Resistance training appears to be a promising strategy owing to its ability to prevent and reverse muscle mass loss.^16^ In contrast, aerobic physical training should be considered to improve these patients’ cardiorespiratory capacity, which becomes impaired as the disease progresses.^17^

However, paradoxical results have been reported in the literature regarding functional capacity in patients with liver cirrhosis who have undergone resistance or aerobic physical training.^18^^,^^19^

Therefore, the literature includes reviews evaluating the effects of physical training on functional capacity in patients with liver cirrhosis^20^ in addition to preventing serious events such as mortality.^21^ However, there is a lack of systematic reviews clarifying the state-of-the-art in relation to the individual or combined effects of resistance and/or aerobic physical training on the quality of life, functional capacity, and body composition of patients with liver cirrhosis.

The present study therefore, aims to conduct a systematic review evaluating the effects of resistance and/or aerobic physical training on quality of life, functional capacity, and body composition in patients with liver cirrhosis.

## Material and methods

### Eligibility criteria

Studies originally published in the following databases were analyzed: MEDLINE via PubMed (National Library of Medicine), Cochrane Library, and Web of Science. Only Randomized Controlled Clinical Trials (RCTs) were analyzed. All trials were approved by institutional ethics committees and declared their compliance with ethical standards, such as the Declaration of Helsinki. To prepare for this systematic review, the authors used the Preferred Reporting Items for Systematic Reviews and Meta-Analyses (PRISMA) systematization and registered it on the PROSPERO platform (CRD42024337202). No data were collected directly from patients, and only previously published randomized controlled trials were included. Therefore, institutional ethics approval and informed consent were not required for this review.

### Search strategies

This study used PICO systematization to formulate the search phrases, utilizing combinations of the following keywords: ‘liver cirrhosis’, ‘exercise’, ‘aerobic exercise’, ‘resistance training’, and their synonyms in the Medical Subject Headings (MeSH) thesaurus. The term ‘randomized controlled trial’ was used to identify eligible study designs. The search was performed by two independent authors (RRG and TCR). Disagreements were resolved by consensus or by referral to the third reviewer (DGM). The inclusion and exclusion criteria and analyzed outcomes are shown in Table 1.

**Table 1:** Inclusion and exclusion criteria and variables analyzed.

**Inclusion criteria**
Design: Randomized controlled clinical trials
Intervention: Resistance and/or aerobic exercises
Only in humans
Adult individuals with liver cirrhosis, regardless of the etiology and severity of the disease
**Exclusion criteria**
Intervention in the control group involving any type of physical training
Publication format: Abstract only
Studies that included some co-intervention in the experimental procedure and/or in the control group
**Analyzed outcomes**
Quality of life ‒ Chronic Liver Disease Questionnaire (CLDQ) and SF-36 Questionnaire
Functional capacity
Body composition (body mass index and thigh circumference)

### Data extraction

The extracted data included study authors, publication year, intervention, control, and analyzed variables such as quality of life, oxygen consumption at peak effort (VO_2_peak), Body Mass Index (BMI), distance covered in the 6-Minute Walk Test (6 MWT), and thigh circumference. If the data were not available, the authors were contacted to retrieve the information. All extractions were cross-checked by a third reviewer, and disagreements were discussed to reach a consensus.

### Effect measures

The meta-analysis was performed using the Review Manager 5.4 program and fixed or random effect statistical analysis. A value greater than or equal to 50 %, as identified by the Higgins test, showed high heterogeneity. A 95 % Confidence Interval (95 % CI) was calculated for each individual study, as well as for the combined estimate across all selected studies.

For the analysis of continuous data, the mean, standard deviation, and sample size were identified for the meta-analysis. For continuous outcomes with the same unit of measurement, the authors used the differences in means as a measure of effect. Differences in standardized means were used for outcomes with different units of measurement. The authors adopted a significance value of *p* < 0.05.

### Risk of bias analysis

To assess the risk of bias in the studies selected for this review, the authors implemented ROB 2.0 software. To assess the risk of publication bias, the authors used Egger’s test with *p* < 0.05. The certainty of the evidence was determined using GRADEpro.^22^

## Results

**Fig. 1:**
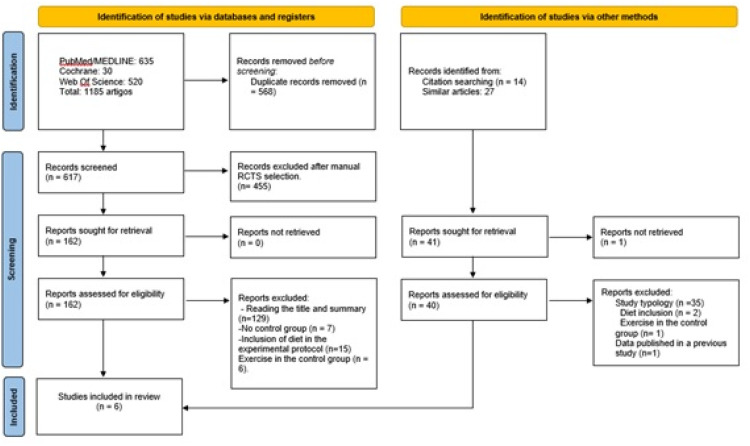
Flowchart of the study selection process.

A total of 1185 studies were identified on physical exercise in patients with liver cirrhosis; however, only six were included in this review after applying the eligibility criteria and excluding duplicate studies. Fig 1 shows the flowchart used to select the articles for analysis.

The studies analyzed involved 244 participants divided into an experimental group with 140 participants and a control group with 104 participants. Participants in the experimental group performed resistance training,^18^^,^^23^ aerobic training^24^, ^25^, ^26^ or combined physical training^19^ and had a mean age of 58.3 ± 3.83 years. Participants in the control group had a mean age of 59.4 ± 2.93 years. Ages ranged from 53 to 63.1 years in both groups. Most of the participants in the selected studies were male; 62 % were in the experimental group, and 71 % were in the control group.

The authors emphasize that 12.75 % of participants in the six included studies did not complete the proposed physical training. They were excluded from the statistical analysis because the per-protocol approach was used.

In general, the selected studies evaluated participants diagnosed with liver cirrhosis classified as Child-Pugh A and B. Only one study evaluated patients classified as Child-Pugh C.^23^ The interventions were performed over durations between 8 and 12 weeks. Studies with aerobic physical training protocols used either a cycle ergometer and/or treadmill, with an exercise duration of 30 min and intensity of 60 %–80 % maximum heart rate,^19^ 60 %–80 % of reserve heart rate,^26^ or 60 %–80 % of VO_2_peak.^24^ Regarding resistance training, studies performed 7–11 types of exercises. This included exercises for the upper and lower limbs (with variable resistance machines and dumbbells), chest, and abdominal exercises (with body resistance). Repetitions ranged from 2 to 4 sets of 10–15 repetitions per muscle group, with the aim of muscle hypertrophy.^18^^,^^19^ One study adopted only elastic resistance and exercises that assisted functional activities.^23^

Participants in the control group were encouraged to continue their usual activities,^23^^,^^24^^,^^26^ muscle relaxation exercises,^25^ and regular routine guidance.^18^^,^^19^

A summary of the studies included in the review is presented in Table 2.

**Table 2:** Summary of the studies selected for the scope of the systematic review.

**Studies**	**Age**	**Sample**	**Intervention**	**Variables Analyzed**				
				**Quality of life**	**VO_2_peak**	**BMI**	**6MWT**	**Thigh circumference**
Aamann L et al., 2020	GE: 61.7 ± 7.8; GC: 63 ± 7.0	GE: *n* = 20 (Child-Pugh *A* = 10, Child-Pugh *B* = 10); GC: *n* = 19 (Child-Pugh *A* = 10; Child-Pugh *B* = 9)	GE: Resistance physical training for 12-weeks; GC: No change in activity level	GE = GC (SF-36)	NA	GE = GC	GE = GC	GE = GC
Lai JC. et al., 2021	GE: 61.3 ± 7.6; GC: 58.3 ± 23.5	GE: *n* = 58 (Child-Pugh *B* = 30, Child-Pugh *C* = 28); GC: *n* = 25 (Child-Pugh *B* = 13; Child-Pugh *C* = 12)	GE: Resistance physical training for 12-weeks; GC: Control (usual care)	GE = GC	NA	NA	NA	NA
Kruger C et al., 2018	GE: 53 ± 0.3; GC: 56.4 ± 8.5	GE: *n* = 20 (Child-Pugh *A* = 14, Child-Pugh *B* = 6); GC: *n* = 20 (Child-Pugh *A* = 14; Child-Pugh *B* = 6)	GE: Aerobic training 8-weeks on a cycle ergometer; 60 % to 80 % of HR reserve 3 × *a* week for 30 min; GC: Usual care	GE = GC	GE = GC	GE = GC	GE > GC	GE = GC
Román E et al., 2016	GE: 62 ± 2.4; GC: 63.1 ± 2.3	GE: *n* = 14 (Child-Pugh *A* = 14); GC: *n* = 9 (Child-Pugh *A* = 9)	GE: Aerobic training 12-weeks (cycle ergometer and treadmill walking 10 to 30 min, 60 % to 70 % of maximum HR; GC: Relaxation exercise	NA	GE = GC	NA	NA	GE = GC
Sirisunhirun P et al., 2022	GE: 55.6 ± 8.9; GC: 57.1 ± 6.7	GE: *n* = 20 (Child-Pugh *A* = 20); GC: *n* = 20 (Child-Pugh *A* = 20)	GE: Aerobic and resistance training 12-weeks, HR 60 % to 80 % of maximum HR 40 min duration; GC: Regular physical activity	GE = GC	NA	GE = GC	GE = GC	GE = GC
Zenith L et al., 2014	GE: 56.4 ± 7.7; GC: 58.6 ± 5.8	Child-Pugh *A* = 14 Child-Pugh *B* = 5	GE: Aerobic training eight weeks (3× per week cycle ergometer 60 % to 80 % of VO_2_peak); GC: Usual care	GE = GC	GE > GC	GE = GC	GE = GC	GE > GC

GE, Experimental Group; GC, Control Group; = Absence of a statistical difference between groups after the intervention (*p* > 0.05); > Statistically significant increase in the variable after the intervention (*p* < 0.05); < Statistically significant decrease in the variable after the intervention (*p* < 0.05); NA, Not evaluated; VO_2_peak, Oxygen consumption at peak effort; BMI, Body Mass Index; 6 MWT, Distance covered in 6-Minute Walk Test.

**Fig. 2:**
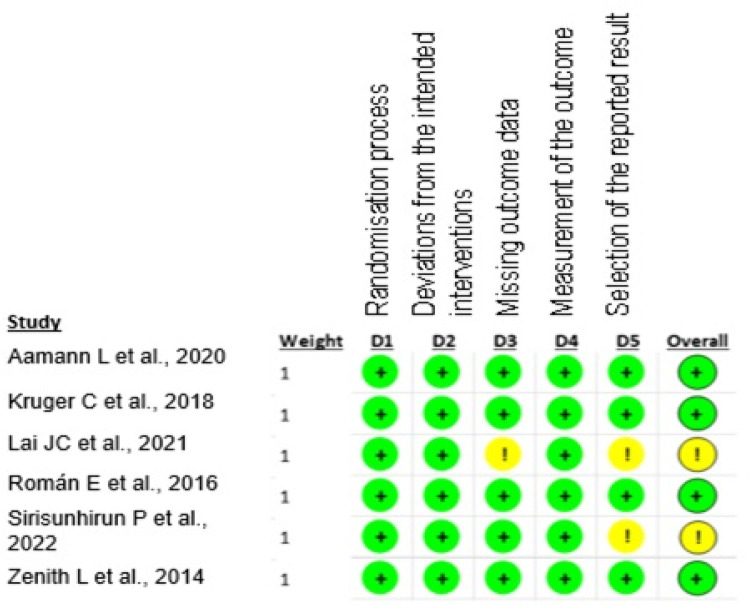
Assessment of the risk of bias in studies selected for the review.

The selected studies presented a low risk of bias, as shown in Fig 2.

**Fig. 3:**
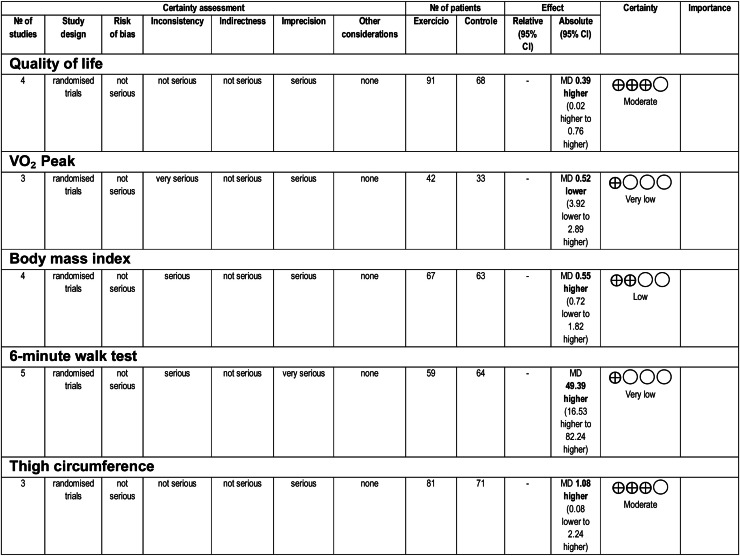
Certainty of the evidence regarding the main analyzed outcomes.

Overall, the certainty of the evidence ranged from very low to moderate. The inconsistency and imprecision of the analyzed results were key factors that lowered the levels of evidence. The certainty of the evidence is shown in Fig 3.

### Quality of life

**Fig. 4:**
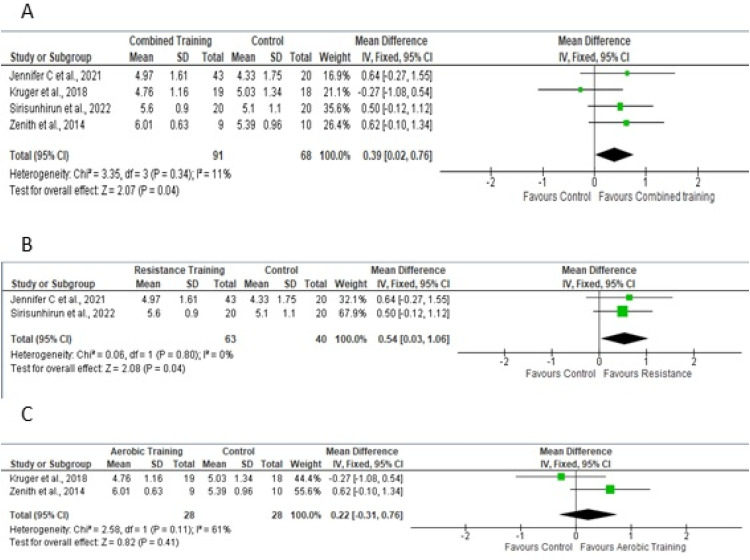
Forest plot of the studies included in the fixed-effect analysis of standardized mean differences for quality of life, with 95 % Confidence Intervals.

For the meta-analysis, four studies^19^^,^^23^^,^^24^^,^^26^ performed aerobic and/or resistance training in the experimental protocol, providing sufficient data to analyze the outcome of quality of life as assessed by the Chronic Liver Disease Questionnaire (CLDQ) scale for the total domain. Fig 4A shows a sample of 159 participants. To measure the effect, the difference in the means was 0.39, adopting a fixed effect. Figs 4B and 4C show the effects of studies that included resistance training in the experimental protocol and only aerobic training on quality of life, respectively.

### Functional capacity

**Fig. 5:**
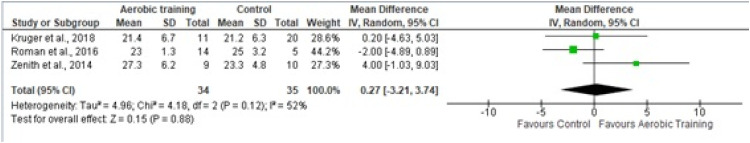
Forest plot of the studies included in the random-effect analysis of the standardized mean differences for functional capacity (VO2peak), with 95 % Confidence Intervals.

Functional capacity was assessed using VO_2_peak and 6 WMT. Three studies used VO_2_peak as a functional capacity outcome, measured by cardiopulmonary testing.^24^, ^25^, ^26^ Only aerobic physical training was used in these experimental protocols, observing an estimated effect, a mean difference of −0.52, and adopting the random effect. The final sample consisted of 69 participants (Fig. 5).

**Fig. 6:**
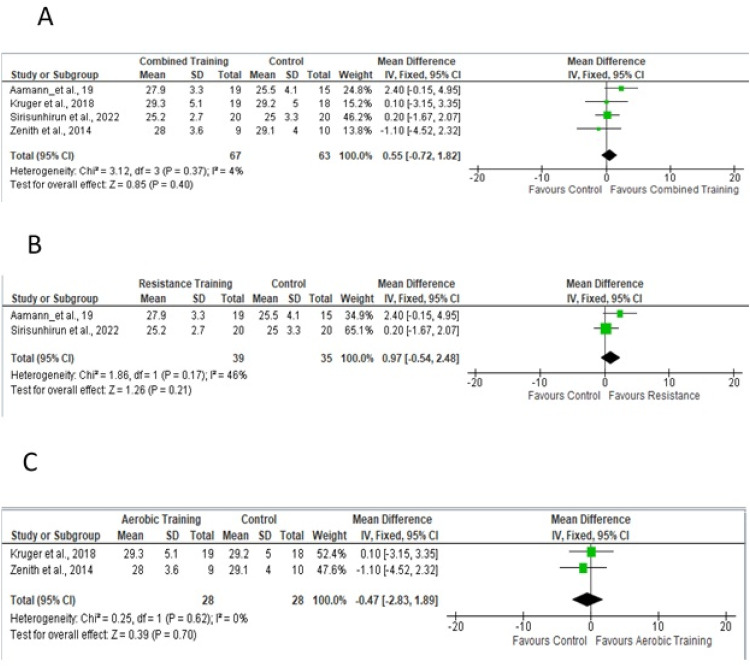
Forest plot of the studies included in the fixed-effect analysis and random-effect analysis of standardized mean differences in 6 WMT, with 95 % Confidence Intervals.

Four studies provided sufficient data for the meta-analysis of 6 WMT.^18^, ^19^, ^20^, ^21^, ^22^, ^23^, ^24^, ^25^, ^26^ Fig 6A shows the studies in which aerobic and/or resistance training was used in the experimental protocol. Fig 6B shows studies that included only resistance training; Fig 6C shows studies that included only aerobic training.

### Body composition

**Fig. 7:**
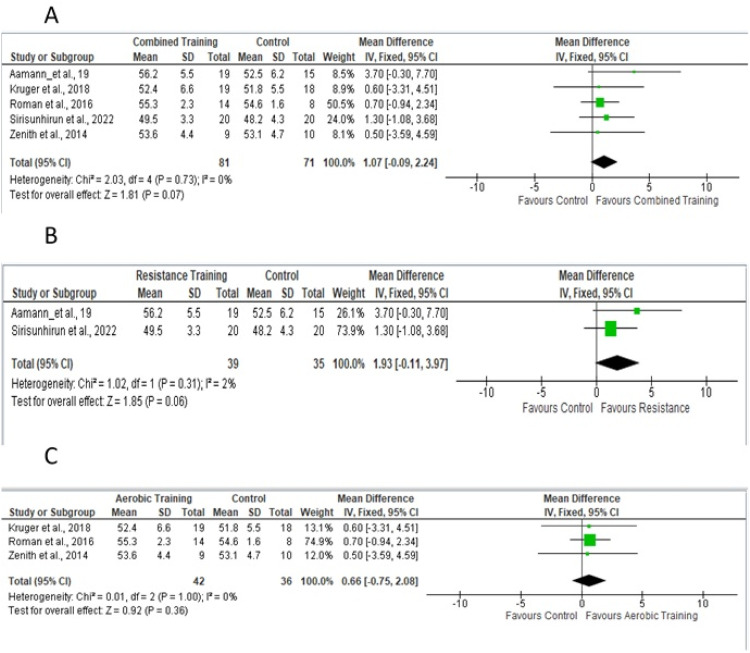
Forest plot of the studies included in the fixed-effect analysis of standardized mean differences for body composition (BMI), with 95 % Confidence Intervals.

Body composition was assessed using BMI and thigh circumference. Four studies^18^^,^^19^^,^^24^^,^^26^ provided sufficient data for the meta-analysis (Fig. 7A) of BMI and aerobic and/or resistance and/or aerobic training. The difference in means for the BMI fixed-effect estimate was −0.55, for a total of 130 participants. Fig 7B shows studies that included resistance training in the experimental protocol; Fig 7C shows studies that included only aerobic training.

**Fig. 8:**
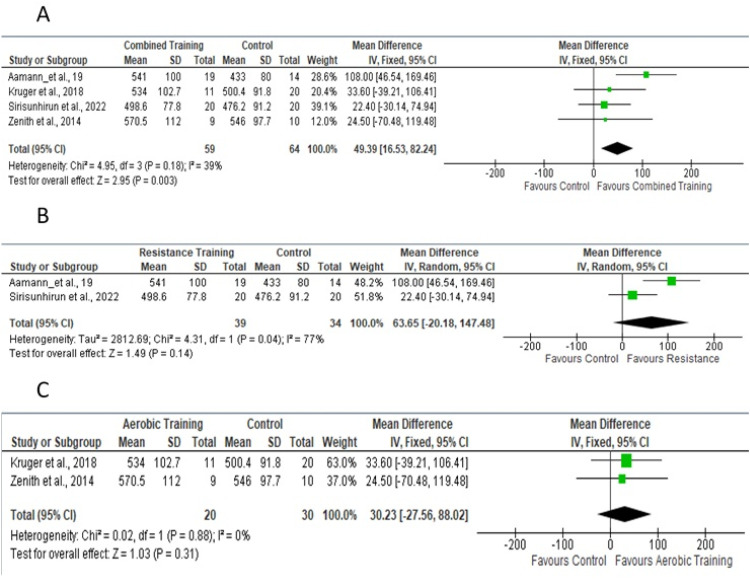
Forest plot of the studies included in the fixed-effect analysis of standardized mean differences for body composition (thigh circumference), with 95 % Confidence Intervals.

Five studies^18^^,^^19^^,^^24^, ^25^, ^26^ were evaluated for the meta-analysis of thigh circumference (Fig. 8A), with a mean fixed-effect difference of 1.08, for a total of 152 participants, which considered the fixed effect of resistance and/or aerobic physical training. Fig 8B shows studies that included resistance training in the experimental protocol; Fig 8C shows studies that included only aerobic training.

## Discussion

The main findings of this review include improved quality of life and performance in the 6 MWT in response to resistance and aerobic physical training. However, no changes were observed in relation to BMI, thigh circumference, or VO_2_peak.

Hepatocyte function is severely impaired in chronic liver disease, which leads to increased intrahepatic resistance and possible portal hypertension.^1^ Research has shown that liver cirrhosis associated with portal hypertension is a known risk factor for sarcopenia.^27^ This reduction in muscle strength can be explained by several mechanisms, such as hormonal dysregulation,^28^ metabolic changes,^29^ a pro-inflammatory state,^30^ insulin resistance,^11^ and increased amino acid catabolism.^31^

Thus, sarcopenia is strongly associated with a worsening quality of life,^32^ and several studies have evaluated the impact of strength training on this outcome.^18^, ^19^, ^20^, ^21^, ^22^, ^23^, ^24^, ^25^, ^26^, ^27^, ^28^, ^29^, ^30^, ^31^, ^32^, ^33^ The results of a meta-analysis^34^ did not demonstrate an improvement or impairment in quality of life after physical training in patients with liver cirrhosis. However, the authors did not evaluate the individual impact of resistance training. Moreover, most studies analyzing this outcome have used aerobic physical training in the intervention group. Thus, in the present review, the authors believe that recent studies of the impact of resistance training^9^^,^^19^ on quality of life may have contributed to changing the understanding of this outcome.

Physical training did not affect the muscle perimeter. This may be because some of the selected studies^24^^,^^26^ used aerobic training in their experimental protocols. Only one study,^18^ exclusively used resistance training, finding a significant increase in the cross-sectional area of the quadriceps muscle and thigh circumference. This is consistent with previous findings that resistance training directly affects muscle hypertrophy.^35^ It is worth mentioning that muscle adaptations to physical training depend on the intensity of the exercise,^36^ the weekly frequency,^37^ and progressive overload stimuli.^38^ These factors may have received insufficient attention in the studies selected for this review, therefore limiting muscle strength gains and generating less impact on the muscle perimeter.

The lack of significant changes in VO_2_peak, BMI, and thigh circumference may be attributed to methodological limitations. Most studies had small sample sizes, which limited their statistical power to detect differences in these outcomes. Furthermore, there was considerable heterogeneity in aerobic and resistance exercise training protocols, especially regarding duration, weekly frequency, intensity, and progression. This probably made it difficult to detect measurable improvements in the outcomes.^39^

Resistance training can increase protein synthesis, stimulate muscle hypertrophy, and consequently, increase lean body mass.^18^ These phenomena can increase ammonia metabolism, which is elevated in patients with liver cirrhosis.^40^ In addition, an increase in lean mass favors the improvement of mitochondrial function in skeletal muscles, providing greater energy generation, improved functional capacity, and quality of life.^24^

Recently, a meta-analysis^20^ demonstrated that functional capacity did not demonstrate positive or negative changes after physical training in patients with cirrhosis classified as Child-Pugh A. However, this review performed an individualized analysis of studies that investigated either aerobic or resistance training to improve functional capacity, as assessed by the 6 MWT. These findings confirm the need to increase resistance training to improve the distance achieved in the test. Resistance training may therefore contribute to improved functional capacity in patients with chronic liver disease.

Although no studies to date have evaluated the minimum clinically important difference in the 6 MWT for patients with liver cirrhosis, the authors report that, physical training groups covered a substantially greater distance than the control groups or adults with other diseases.^41^

Notably, no study selected for this review reported adverse events during resistance and aerobic physical training. However, it has been observed that aerobic exercise, even at low intensities, can acutely cause an acute increase in portal pressure.^42^ Nevertheless, aerobic physical training associated with strength training leads to a long-term reduction in portal pressure in patients with chronic liver disease compared with their peers who did not perform physical training.^43^ This can be explained by improved endothelial function associated with a reduction in the inflammatory cascade and a decrease in the renin-angiotensin and aldosterone systems, leading to lower intrahepatic resistance and, consequently, a decrease in hepatic portal pressure.^44^

From a clinical perspective, most studies included in this review enrolled patients classified as Child-Pugh class A or B. Following a period of physical training, these patients demonstrated improvements in functional capacity and quality of life, with no reported adverse events. Although an optimal protocol has not yet been established, exercise programs lasting 8–12 weeks (2–3 sessions per week) and incorporating both aerobic and resistance training have been shown to be effective in improving these outcomes. Exercise prescriptions should include gradual progression and individualized adjustments, especially during resistance training, to ensure safety and maximize effectiveness. For patients with advanced cirrhosis (Child-Pugh class C), there is currently insufficient evidence on the potential benefits of exercise prescriptions. Caution and clinical monitoring are recommended until more robust data are available.

Furthermore, the progression of exercise protocols is inadequately reported, especially in resistance training programs. This limits the practical applicability of the findings from these studies, making it difficult to replicate reportedly effective interventions in clinical practice.

The practical implications of the present findings are summarized in Supplementary Table 1, which provides evidence-based recommendations to guide exercise interventions in patients with liver cirrhosis.

This review makes an original contribution by separately evaluating the effects of aerobic and resistance training in patients with cirrhosis. In particular, resistance training may improve patient quality of life. It also revealed methodological gaps in existing literature, such as the lack of standardized training protocols and the underrepresentation of patients with advanced liver disease (Child-Pugh class C).

The certainty of the evidence ranged from very low to moderate, which limits the strength of the conclusions and emphasizes the need for future clinical trials with greater methodological rigor. These findings highlight the importance of standardized interventions, larger sample sizes, and patient-centered outcomes to enhance the clinical applicability and safety of research in this population.

## Conclusions

Aerobic and resistance training appear to improve quality of life and functional capacity without altering body composition in patients with liver cirrhosis. However, these results should be interpreted with caution, considering the methodological limitations and heterogeneity of the included studies. Furthermore, owing to the very low and moderate certainty of the evidence, new controlled and randomized clinical trials with aerobic and/or resistance training in patients with liver cirrhosis should be conducted to confirm these results. Future studies should also prioritize the use of standardized exercise protocols to enhance comparability and reproducibility.

The practical implications of these findings are summarized in Supplementary Table 1, which provides evidence-based recommendations to guide exercise interventions in patients with liver cirrhosis.

## CRediT authorship contribution statement

**Thiago Casali Rocha:** Conceptualization, Methodology, Software, Validation, Formal analysis, Writing – original draft, Writing – review & editing. **Rafael Ribeiro Germano:** Investigation, Data curation, Writing – original draft. **Maria Clara Dias Giacomini:** Visualization, Investigation, Writing – original draft. **Fábio Heleno de Lima Pace:** Supervision, Conceptualization. **Mateus Camaroti Laterza:** Conceptualization, Methodology, Validation, Writing – original draft. **Daniel Godoy Martinez:** Conceptualization, Methodology, Validation, Writing – original draft, Writing – review & editing.

## Declaration of competing interest

The authors declare no conflicts of interest.

## Appendix Supplementary materials

Image, application 1
