# Supplementary material for: Effect of aerobic and resistance training on body composition, functional capacity, and quality of life in patients with liver cirrhosis: a systematic review with meta-analysis
Source: Clinics (Sao Paulo). 2025 Sep 20;80:100748. doi: 10.1016/j.clinsp.2025.100748 (PMC12489835; doi:10.1016/j.clinsp.2025.100748)
Supplement: Supplementary file 1 [file mmc1.docx]

**CLINICS-D-25-00167_ Supplementary Material**

**Supplementary Table 1** Practical recommendations based on review findings.

**Target Population:**

Patients with liver cirrhosis classified as Child-Pugh class A or B.

**Intervention Duration:**

Training programs lasting from 8- to 12-weeks.

| **Aerobic Exercise Evidence-Based Recommendations** | |
| --- | --- |
| Frequency | 2–3 sessions per week |
| Intensity | 60%–80% of maximum heart rate, 60%–80% heart rate reserve, or 60%–80% VO_2_peak |
| Time | 30 minutes per session |
| Type | Cycle ergometer, treadmill, or supervised/home walking |
| **Resistance Exercise Evidence-Based Recommendations** | |
| Frequency | 2–3 sessions per week |
| Intensity | Progressive individualized overload (moderate to high) |
| Time | No specific duration |
| Type | Exercises using free weights, machines, body weight, or elastic bands |
| Repetitions | 10–15 repetitions |
| Sets | 2–4 sets |
